# New species and new records of Trigonalyidae (Hymenoptera) from Tibet, China

**DOI:** 10.3897/zookeys.918.49729

**Published:** 2020-03-12

**Authors:** Hua-Yan Chen, Chun-Dan Hong, Cornelis van Achterberg, Hong Pang

**Affiliations:** 1 State Key Laboratory of Biocontrol, School of Life Sciences / School of Ecology, Sun Yat-sen University, Guangzhou 510275, China Sun Yat-sen University Guangzhou China; 2 Bureau of Agriculture and Rural Affairs of Longhu, Shantou 515000, China Bureau of Agriculture and Rural Affairs of Longhu Shantou China; 3 State Key Laboratory of Rice Biology and Ministry of Agriculture / Key Lab of Agricultural Entomology, Institute of Insect Sciences, Zhejiang University, Hangzhou 310058, China Zhejiang University Hangzhou China

**Keywords:** Hyperparasitoid, *
Jezonogonalos
*, *
Orthogonalys
*, parasitoid, *
Taeniogonalos
*, *
Teranishia
*, trigonalid wasp

## Abstract

Two new species of Trigonalyidae are described from Tibet (SW China): *Jezonogonalos nyingchiensis* Chen & van Achterberg, **sp. nov.**, and *Taeniogonalos eurysoma* Chen & van Achterberg, **sp. nov.** In total, seven species representing four genera are known from Tibet, and two of them are newly recorded from Tibet: *Taeniogonalos bucarinata* Chen, van Achterberg, He & Xu, 2014, and *Teranishia crenulata* Chen, van Achterberg, He & Xu, 2014.

## Introduction

Trigonalyidae is a small family of Hymenoptera in its own superfamily Trigonalyoidea, with approximately 120 recognized species in 16 genera worldwide (Carmean and Kimsey 1998; Smith and Stocks 2005; Santos et al. 2012; Smith and Tripotin 2012; Smith et al. 2012; Chen et al. 2014; Yamane 2014; Smith and Tripotin 2015; Tan et al. 2017; Lelej 2019). The family name Trigonalidae and Trigonlyidae have been used by different authors, but we follow Weinstein and Austin (1991) and Lelej (2003) in using the family name as corrected by Krieger (1894) to Trigonalyidae; for the argumentation see Lelej (2003), and Engel and Lelej (2020).

The biology of trigonalid wasps is peculiar. Rather than laying their eggs directly on or in their host, females of these wasps lay thousands of minute eggs on foliage, which must be eventually consumed by caterpillars or sawfly larvae. Once inside the caterpillars or sawfly larvae, the wasp egg either hatches and attacks any other parasitoid larvae (wasps: Ichneumonidae or Braconidae; flies: Tachinidae) or it waits until the caterpillars or sawfly larvae are fed to a Vespidae larva, which it then attacks. Therefore, these wasps are hyperparasitoids or primary parasitoids, but extremely unusual among hymenopterans (Carmean and Kimsey 1998; Murphy et al. 2009).

The greatest diversity of this family occurs in tropical and subtropical regions. In fact, the family seems to be absent from arctic and alpine habitats (Carmean and Kimsey 1998), though they were found to be fairly common at 1300–1500 m altitude in the Qinling Mountains of NW China (Tan et al. 2017). Here we describe two new species and record two described species from the mountainous province of Tibet.

## Material and methods

This work is based upon specimens in the following collections, with abbreviations used in the text: **SYSBM**, Sun Yat-sen University, The Museum of Biology, Guangzhou, China; **ZJUH**, Institute of Insect Sciences, Zhejiang University, Hangzhou, China. Morphological terminology generally follows Chen et al. (2014). Images and measurements were made using a Nikon SMZ25 microscope with a Nikon DS-Ri 2 digital camera system. Images were post-processed with Adobe Photoshop CS6 Extended. YPT stands for collected in yellow pan trap.

## Taxonomy

### Jezonogonalos

Taxon classificationAnimaliaHymenopteraTrigonalyidae

Tsuneki, 1991

1A396ACB-FD85-50BB-B92D-AAE64A42756A

Figs 1–3 , 4–11 , 12–14 , 15–22

Jezonogonalos Tsuneki, 1991: 32, 2003: 4; Carmean and Kimsey 1998: 70; Chen et al. 2014: 22–44 (diagnosis, key). Type species: Jezonogonalos marujamanae Tsuneki, 1991 [= J. marujamae Tsuneki, 1991], by monotypy. Synonymized with Pseudogonalos Schulz, 1906, by Lelej (1995) and re-instated by Chen et al. (2014).

#### Diagnosis.

Antenna black and with 23–27 segments; area above supra-antennal elevations flat, more or less punctate, without protuberance between elevations and inner side of supra-antennal elevations flat, smooth and black; tyloids of male antenna present on 10^th^–16^th^ segments, short and nearly circular or elliptical; occipital carina widened medio-dorsally; apical segment of labial palp widened and obtuse, more or less triangular; vertex normal, at most with slight median depression dorsally; mandibles wide in anterior view and sublaterally attached to head; metanotum strongly convex and finely sculptured medially; anterior propodeal sulcus crenulate and medially widened; posterior propodeal carina curved and distinctly protruding and more or less separated from foramen medio-dorsally; fore wing with large dark patch below pterostigma; vein 1-SR of fore wing long; hind trochanter black or ivory; hind tarsus slightly or not modified; second and third sternites of female flat and moderately sclerotized and no protuberances; body without pale pattern, at most malar space and margins of basal metasomal sternites and tergites narrowly ivory, remainder black (Chen et al. 2014).

#### Biology.

Unknown. Collected in June–November.

#### Distribution.

China, Japan. Before this study, eight species of this genus had been described from China, with only one species recorded from Tibet. We describe here another species new to science from Tibet.

### Jezonogonalos jiangliae

Taxon classificationAnimaliaHymenopteraTrigonalyidae

Chen, van Achterberg, He & Xu, 2014

129C8606-B549-5F96-8B53-9884D25EEC33

Figs 1–3 , 4–11

Jezonogonalos jiangliae Chen et al., 2014: 29–32 (diagnosis, description, distribution).

#### Material examined.

1 male, China: Tibet, Nyingchi, Yigong, 225 6m, 30°10'53"N, 94°54'30"E, 3.viii.2018, sweep net, SCAU 3040486 (SYSBM); 3 males, China: Tibet, Nyingchi, Yigong, Tongjiacun, 2214 m, 30°14'12"N, 94°53'48"E, 6.viii.2018, YPT, SCAU 3040489, SCAU 3040187, SCAU 3040188 (SYSBM).

#### Distribution.

China (Tibet). Collected at 2214–2256 m.

#### Notes.

*Jezonogonalos jiangliae* was first described by Chen et al. (2014) based on a single male without complete antennae from Tibet. Based on the additional material, this species shows the following variations: male antenna with 25 or 26 segments, with tyloids present on 10^th^–15^th^ or 11^th^–16^th^ segments; clypeus usually entirely black, but sometimes partly ivory; second tergite sometimes with ivory spots latero-posteriorly. The female of this species is still unknown. As Chen et al. (2014) suggested, collection at the type locality and the use of COI (“barcoding”) will recover the conspecific female.

**Figures 1–3.**
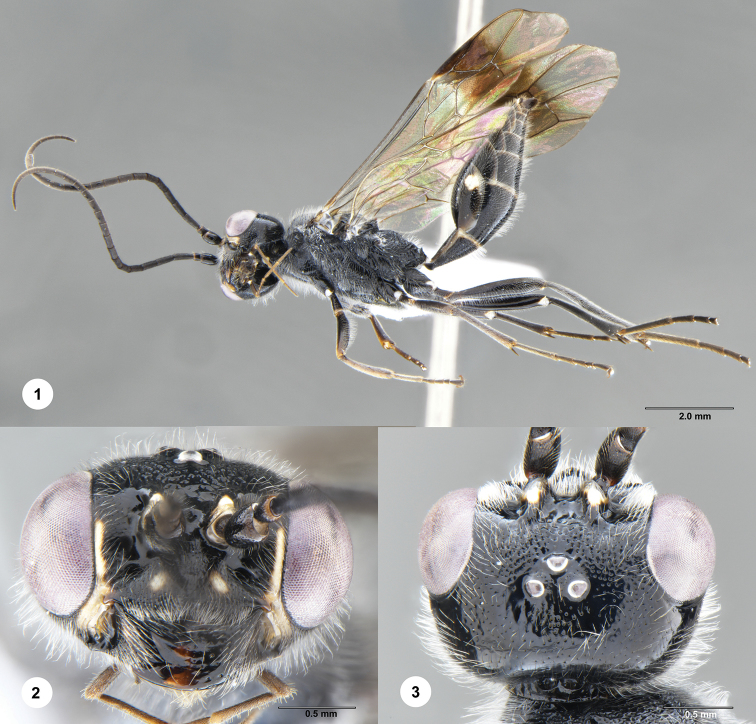
*Jezonogonalos jiangliae* Chen, van Achterberg, He & Xu, male (SCAU 3040188). **1** Habitus, lateral aspect **2** head, anterior aspect **3** head, dorsal aspect.

**Figures 4–11.**
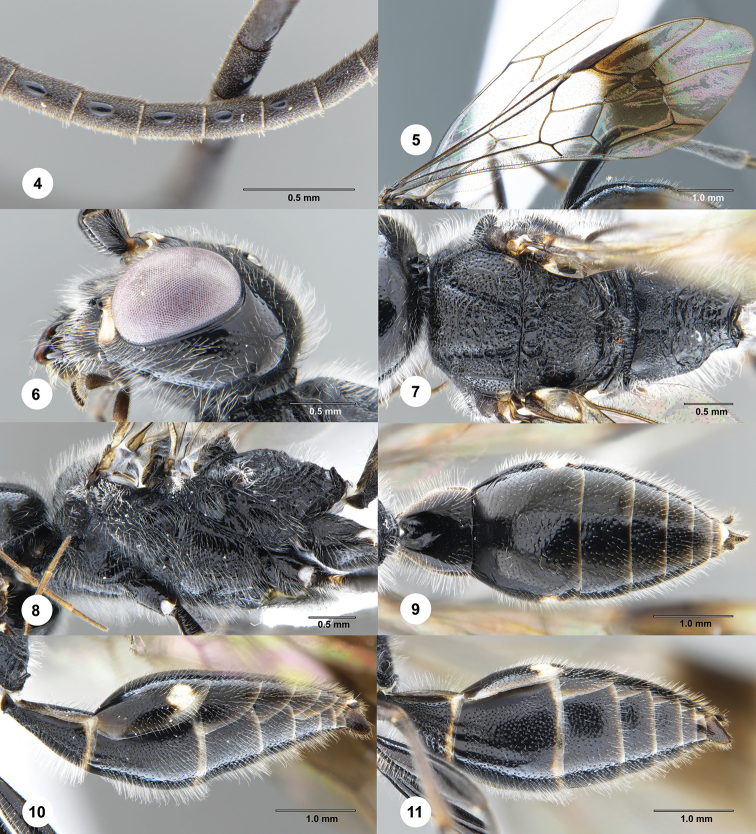
*Jezonogonalos jiangliae* Chen, van Achterberg, He & Xu, male (SCAU 3040188). **4** Tyloids on 11^th^–15^th^ segments of antenna **5** wings **6** head, lateral aspect **7** mesosoma, dorsal aspect **8** mesosoma, lateral aspect **9** metasoma, dorsal aspect **10** metasoma, lateral aspect **11** metasoma, latero-ventral aspect.

### Jezonogonalos nyingchiensis

Taxon classificationAnimaliaHymenopteraTrigonalyidae

Chen & van Achterberg sp. nov.

42D18857-DDCD-53AB-9058-154480957B04

http://zoobank.org/C47806BA-246D-4131-B4F4-8B632009CD26

Figs 12–14 , 15–22

#### Material examined.

***Holotype***, female, China: Tibet, Nyingchi, Yigong, 2256 m, 30°10'53"N, 94°54'30"E, 3.viii.2018, sweep net, SCAU 3040487 (deposited in SYSBM). ***Paratypes***: 2 females, same data as holotype.

#### Diagnosis.

Occipital carina very wide medio-dorsally, with pair of curved lamellae separated by a carina (Fig. 14); outer side of supra-antennal elevations subvertical, smooth, and elevations approximately 0.6 × as long as scapus (Fig. 14); frons densely punctate dorsally and laterally, largely smooth ventrally and medially (Fig. 13); supra-antennal elevations largely ivory dorsally (Fig. 14); mandible mainly black, except dark brown base of teeth (Fig. 13); metasoma dorsally largely smooth and largely black (Fig. 20); first tergite approximately 0.7 × as long as its apical width (Fig. 20); third sternite approximately 0.4 × as long as second sternite (Fig. 22).

**Figures 12–14.**
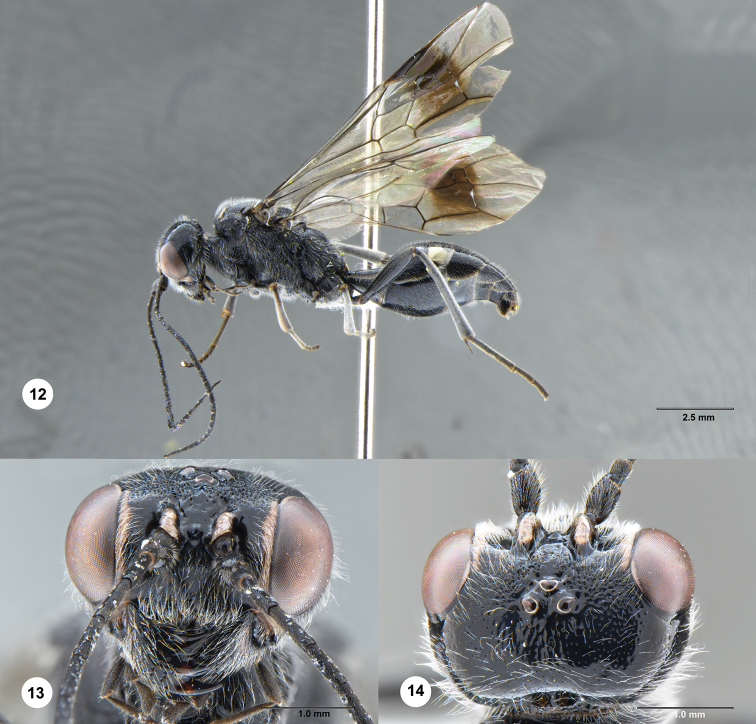
*Jezonogonalos nyingchiensis* Chen & van Achterberg, sp. nov., holotype, female (SCAU 3040487). **12** Habitus, lateral aspect **13** head, anterior aspect **14** head, dorsal aspect.

**Figures 15–22.**
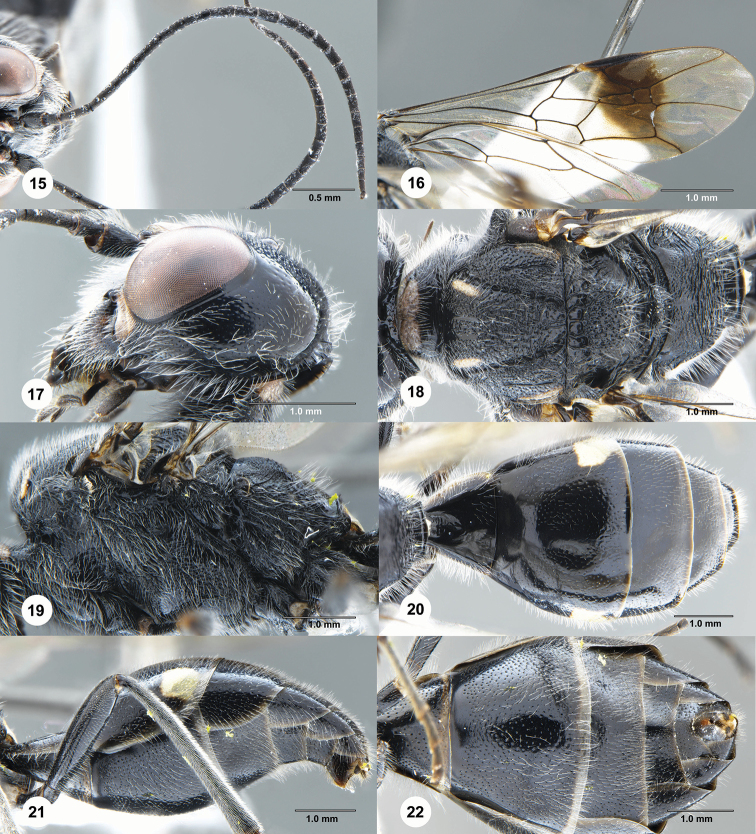
*Jezonogonalos nyingchiensis* Chen & van Achterberg, sp. nov., holotype, female (SCAU 3040487). **15** Antenna **16** wings **17** head, lateral aspect **18** mesosoma, dorsal aspect **19** mesosoma, lateral aspect **20** metasoma, dorsal aspect **21** metasoma, lateral aspect **22** metasoma, ventral aspect.

#### Comments.

This species is similar to *J. shaanxiensis* from Shaanxi (NW China) and it would run to that taxon (couplet 7) in the key of Tan et al. (2017), but can be distinguished by having the frons largely smooth medially and the mesopleuron mainly punctate-rugose, but narrowly smooth posteriorly.

#### Description.

Holotype, ♀, length of body 11.8 mm (of fore wing 9.8 mm).

***Head.*** Antenna with 25 segments; frons densely punctate dorsally and laterally, with medium-sized whitish setae, largely smooth ventrally and medially (Fig. 13); vertex largely smooth, moderately punctulate behind posterior ocellus (Fig. 14); temple largely smooth, punctulate (Fig. 17); head hardly narrowed behind eyes, eye in dorsal view 1.1 × as long as temple (Fig. 14); occipital carina strongly widened and pair of circular lamellae medio-dorsally, separated by a distinct carina (Figs 14, 17), laterally distinctly crenulate; supra-antennal elevations distinctly enlarged (approximately 0.6 × as long as scapus), smooth and outer side subvertical; clypeus concave and thick medio-ventrally and area above it convex and obtusely protruding (Fig. 13).

***Mesosoma.*** Mesosoma 1.7 × as long as its dorsoventral height (Fig. 19); mesopleuron mainly punctate-rugose, narrowly smooth posteriorly; notauli wide, deep and coarsely crenulate; middle lobe of mesoscutum smooth antero-medially, otherwise transversely punctate-rugose, lateral lobes mainly punctate except for a smooth line (Fig. 18); scutellar sulcus wide and coarsely crenulate; scutellum coarsely punctate and laterally with smooth spaces between longitudinal rugae, flattened, in lateral view below level of metanotum (Fig. 18); metanotum medially protruding, obtuse and densely and finely punctate (Fig. 18); propodeum antero-laterally irregularly rugulose to smooth, remainder coarsely transversely rugose and shiny medially, and smooth posteriorly (Fig. 18); posterior propodeal carina thick lamelliform (foramen approximately 4 × as wide as high medially).

***Wings.*** Fore wing: length of vein 1-M 1.4 × as long as vein 1-SR; third submarginal cell much wider anteriorly than petiolate second cell (Fig. 16).

***Metasoma.*** First tergite 0.7 × as long as its apical width, smooth but basal depression anteriorly with some crenulae (Fig. 20); second and following tergites shiny and smooth except for punctulation; sternites rather sparsely finely punctate, with wide smooth interspaces; second sternite weakly curved in lateral view; third sternite approximately 0.4 × as long as second sternite (Fig. 22); hypopygium triangularly protruding in ventral view (Fig. 21).

***Colour.*** Black; inner orbita narrowly ivory and connected to ivory malar space; pair of faint patches on clypeus, basal patch of mandible, large patch on supra-antennal elevations, large patch on anterior margin of pronotum, pair of elongate patches on middle lobe of mesoscutum anteriorly, pair of narrow lines near tegulae, epipleura of tergites, large patch apico-laterally on second tergite and narrow apical bands of sternites ivory; mandible teeth dark brown basally (Fig. 13); tegulae mainly dark brown; palpi dark brown; legs mainly black, but fore femur apico-ventrally brownish; pterostigma basally yellow, and remainder dark brown; large area below pterostigma dark brown and remainder of wing membrane subhyaline (Fig. 18).

***Variations.*** Length of body 10.8–11.2 mm, of fore wing 8.9–9.4 mm; metanotum black or with pair of faint ivory spots medially; ivory patches of clypeus and mesoscutum rather small to large; length of vein 1-M of fore wing 1.3–1.5 × as long as vein 1-SR.

**Male.** Unknown.

#### Biology.

Unknown.

#### Distribution.

China (Tibet). Collected at 2256 m.

#### Etymology.

Named after Nyingchi County, where it was collected. Treat as an adjective in apposition.

### Orthogonalys

Taxon classificationAnimaliaHymenopteraTrigonalyidae

Schulz, 1905

1F5712F7-2789-5898-8995-ECF2477456AF

Orthogonalys Schulz, 1905: 76; Weinstein and Austin 1991: 421; Carmean and Kimsey 1998: 52; Smith and Tripotin 2012: 3; Chen et al. 2014: 60–87 (synonymy, diagnosis, key to Chinese species). Type species: Orthogonalys boliviana Schulz, 1905, by monotypy.

#### Diagnosis.

Antenna with 21–32 segments, often with a pale band in apical third of antenna and slender medially; male antenna without tyloids; supra-antennal elevations smooth and shiny, usually comparatively large, without depression dorsally and moderately to widely separated; vertex normal, at most with slight median depression dorsally; apical segment of labial palp widened and obtuse, more or less triangular; mandibles wide in anterior view and sublaterally attached to head; occipital carina usually narrow and smooth; mesoscutum and scutellum often smooth or sparsely punctulate, at most moderately punctate with wide smooth interspaces; metanotum concave latero-dorsally and often sculptured, matt and distinctly convex medially; anterior propodeal sulcus distinctly crenulate, rarely partly reduced; posterior propodeal carina curved and lamelliform; vein 1-SR of fore wing medium-sized to long; fore wing subhyaline, at most slightly infuscate below pterostigma in female; triangular dorso-apical part of hind trochanter separated by an oblique groove; fore trochanter subparallel-sided and distinctly longer than hind trochanter; hind tarsus slightly or not modified; second metasomal sternite and tergite flat in lateral view, weakly sclerotized and smooth; second sternite in ventral view flat medially or weakly convex and no medial elevation or teeth posteriorly; basal half of third sternite flat, without a distinct ledge anteriorly; fifth sternite of female straight or slightly emarginate medio-posteriorly; body often slender (including metasoma) and sometimes ichneumonid-like (Chen et al. 2014).

#### Biology.

Reared as hyperparasitoid of Tachinidae in caterpillars of the family Limacodidae (Carmean and Kimsey 1998; Murphy et al. 2009). Collected in May–August.

#### Distribution.

Mainly East Palaearctic and Northeast Oriental regions, with a few species in East Afrotropical (including Madagascar), Neotropical and Nearctic regions. Chen et al. (2014) and Tan et al. (2017) reported eight species of *Orthogonalys* from China, with only one species from Tibet.

### Orthogonalys elongata

Taxon classificationAnimaliaHymenopteraTrigonalyidae

(Teranishi, 1929)

81EE3087-9B25-5E9C-BB9D-2323419BAB78

Orthogonalos elongata Teranishi, 1929: 146; Marshakov 1981: 105; Tsuneki 1991: 20; Weinstein and Austin 1991: 424.
Satogonalos elongata ; Weinstein and Austin 1991: 424.
Orthogonalys elongata ; Carmean and Kimsey 1998: 54; Bennett and Lelej 2003: 8; Chen et al. 2014: 62, 72–80 (key, synonymy, diagnosis, description, distribution); Tan et al. 2017: 37, 39 (key, distribution).

#### Material examined.

4 females, China: Tibet, Motuo, 13.vii.2013, Zhen Liu, 201300022–201300025 (ZJUH).

#### Distribution.

China (Henan, Shaanxi, Sichuan, Tibet); Russia (South Sakhalin, South Kurils); Japan (Hokkaido, Honshu).

### Taeniogonalos

Taxon classificationAnimaliaHymenopteraTrigonalyidae

Schulz, 1906

4BD57DCF-943F-5190-9E89-6614301EADAD

Figs 23–25 , 26–33

Taeniogonalos Schulz, 1906: 212; Weinstein and Austin 1991: 416; Tsuneki 1991: 59; Carmean and Kimsey 1998: 65; Chen et al. 2014: 95–193 (synonymy, references, diagnosis, key to Chinese species). Type species: Trigonalys maculata Smith, 1851, by monotypy.

#### Diagnosis.

Antenna with 21–26 segments, without pale band and slender medially; male antenna with linear tyloids (= elevated elongate areas) on 11^th^–16^th^ antennal segments; supra-antennal elevations smooth or punctate, without depression dorsally, remain far separated from each other medially and without horizontal “shelf” between antennal bases; temple usually punctate or reticulate-punctate and moderately shiny; occipital carina ending at hypostomal carina at level of mandibular base; vertex flattened, without median depression dorsally; apical segment of labial palp widened and obtuse, more or less triangular; mandibles wide in anterior view and sublaterally attached to head; mesoscutum and scutellum distinctly punctate or rugose; metanotum at least partly convex latero-dorsally and often sculptured; vein 1-SR of fore wing medium-sized to long; fore wing often with subapical dark patch or large part of fore wing dark brown; triangular dorso-apical part of hind trochanter separated by an oblique groove; fore trochanter subparallel-sided and distinctly longer than hind trochanter; hind tarsus slightly or not modified; propodeal foramen more or less arched dorsally and often with a lamelliform carina; second sternite convex in lateral view (but less so in males), strongly sclerotized and frequently densely punctate, sometimes with a medio-posterior elevation but without pair of small teeth; basal half of third sternite flat, without a distinct ledge anteriorly; hypopygium of female pointing anteriorly toward second sternite or straight down or pointing posteriad (Chen et al. 2014).

**Figures 23–25.**
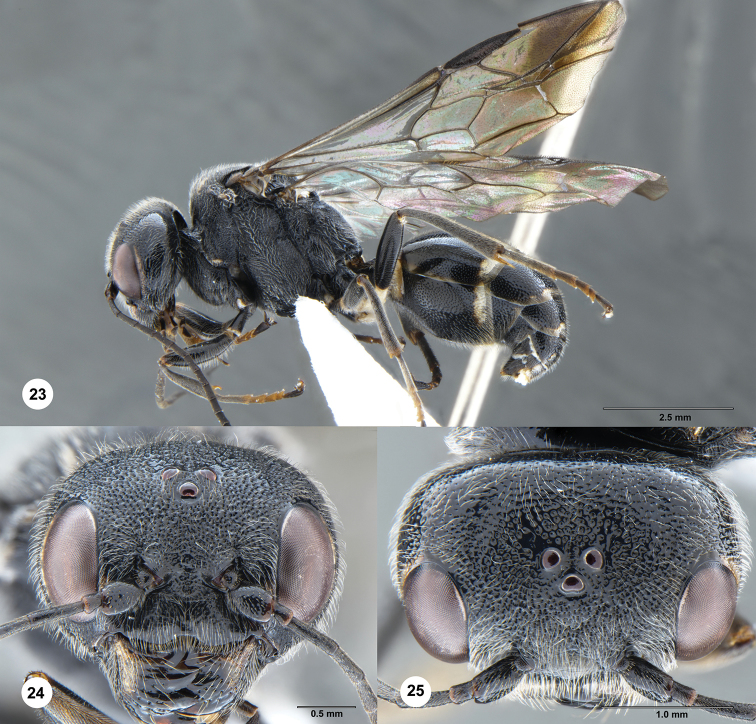
*Taeniogonalos eurysoma* Chen & van Achterberg, sp. nov., holotype, female (SCAU 3040488). **23** Habitus, lateral aspect **24** head, anterior aspect **25** head, dorsal aspect.

#### Biology.

Reared as hyperparasitoid of parasitoid wasps (Ichneumonidae and Braconidae) and parasitoid flies (Tachinidae) in caterpillars, but some species are primary parasitoids of pergid sawflies in Australia (Raff 1934; Carne 1969; He and Chen 1986; Weinstein and Austin 1995; Carmean and Kimsey 1998). Collected mainly in April–October, rarely in November or January.

#### Distribution.

This genus occurs in all major regions, but is unknown from Europe and western Nearctic region. Most of the species occur in the East Palaearctic, Northeast Oriental, and Neotropical regions (Carmean and Kimsey 1998). Chen et al. (2014) reported two species (*Taeniogonalos formosana* (Bischoff 1913) and *T. taihorina* (Bischoff 1914)) from Tibet. Here we describe a third species new to science and report a fourth species from this region.

### Taeniogonalos bucarinata

Taxon classificationAnimaliaHymenopteraTrigonalyidae

Chen, van Achterberg, He & Xu, 2014

508B1CF1-5FE3-5D22-9FD7-5E92F5DE37AA

Taeniogonalos bucarinata Chen, van Achterberg, He & Xu, 2014: 108–113 (description, diagnosis, distribution); Tan et al. 2017: 52–53 (distribution).

#### Material examined.

1 male, China: Tibet, Yadong County, Renqinggang Village, 3083 m, 18.vii.2013, Zhen Liu, 201300035 (ZJUH); 1 male, China: Tibet, Nielamu County, 26.vii.2013, Zhen Liu, 201300123 (ZJUH).

#### Distribution.

China (Fujian, Gansu, Henan, Ningxia, Shaanxi, Sichuan, Tibet, Yunnan, Zhejiang). Collected at 1200–3083 m.

#### Note.

This species is newly recorded from Tibet.

### Taeniogonalos eurysoma

Taxon classificationAnimaliaHymenopteraTrigonalyidae

Chen & van Achterberg sp. nov.

1FFEF4B6-758D-527E-8C49-7C8DA6D5C907

http://zoobank.org/8806BD55-3128-4E08-942D-44F6FD92D04A

Figs 23–25 , 26–33

#### Material examined.

***Holotype***, female, China: Tibet, Nyingchi, Yigong, 2268 m 30°15'10"N, 94°48'24"E, 5.viii.2018, sweep, SCAU 3040488 (deposited in SYSBM). ***Paratype***: 1 female China: Tibet, Nyingchi, Yigong, 2256 m, 30°10'53"N, 94°54'30"E, 3.viii.2018, sweep net (SYSBM).

#### Diagnosis.

Supra-antennal elevations medium-sized (approximately 0.2 × as long as scapus) and their outer side oblique (Fig. 25); occipital carina narrow, non-lamelliform, smooth (Fig. 25); head anteriorly and posteriorly, and pronotum laterally entirely black (Figs 24, 25, 28); vertex reticulate-punctate behind stemmaticum and near eyes, becoming spaced punctate (with interspaces much wider than width of punctures) posteriorly (Fig. 25); mesoscutum coarsely sculptured (Fig. 29); notauli wide and crenulate (Fig. 29); scutellum coarsely rugose, convex laterally and shallowly concave medially (Fig. 29); metanotum slightly convex, rugose (Fig. 29); posterior propodeal carina distinctly arched, narrow lamelliform, foramen comparatively narrow (Fig. 29); posterior margin of tergites, 1^st^ and 2^nd^ sternites with ivory stripes (Figs 31–33); second sternite slightly convex (Fig. 32); third sternite without depression (Fig. 33).

**Figures 26–33.**
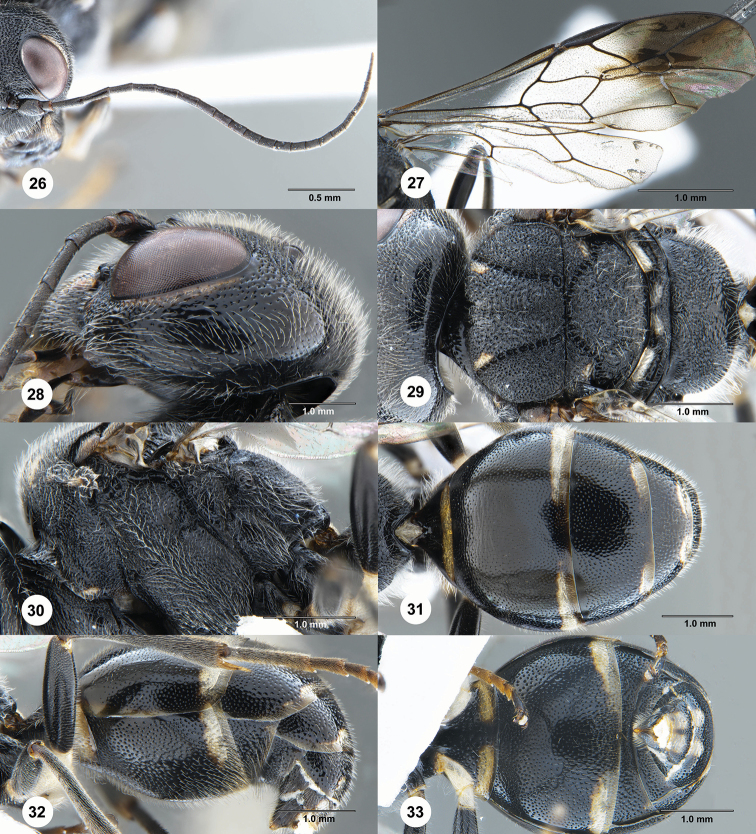
*Taeniogonalos eurysoma* Chen & van Achterberg, sp. nov., holotype, female (SCAU 3040488). **26** Antenna **27** wings **28** head, lateral aspect **29** mesosoma, dorsal aspect **30** mesosoma, lateral aspect **31** metasoma, dorsal aspect **32** metasoma, lateral aspect **33** metasoma, ventral aspect.

#### Comments.

This species is close to *T. alticola* and it would run to that taxon (couplet 17) in the revised key of Tan et al. (2017), but can be distinguished by having smaller supra-antennal elevations, notauli wide, deep and coarsely crenulate, and wide metasomal segments.

#### Description.

Holotype, female, length of body 8.7 mm (of fore wing 7.8 mm).

***Head.*** Antenna with 25 segments; frons reticulate-punctate (Fig. 24); vertex reticulate-punctate behind stemmaticum and near eyes, becoming spaced punctate (interspaces much wider than width of punctures) posteriorly (Fig. 25); temple largely smooth with few punctures at orbita and densely punctate near mandible (Fig. 28); head gradually narrowed behind eyes, eye in dorsal view 0.9 × as long as temple (Fig. 25); occipital carina narrow, non-lamelliform, smooth (Fig. 25); supra-antennal elevations medium-sized (approximately 0.2 × as long as scapus) and their outer side oblique (Fig. 25); clypeus distinctly concave and thick medio-ventrally (Fig. 24).

***Mesosoma.*** Mesosoma 1.5 × as long as its dorso-ventral height (Fig. 30); mesopleuron largely punctate-rugose, becoming densely punctate posteriorly; transverse mesopleural groove narrow, crenulate; notauli wide, deep and coarsely crenulate; middle lobe of mesoscutum smooth antero-medially, otherwise transversely punctate-rugose, lateral lobes densely punctate anteriorly, becoming punctate-rugose posteriorly (Fig. 29); scutellar sulcus complete, moderately narrow and crenulate; scutellum coarsely rugose, convex laterally and shallowly concave medially, anteriorly distinctly above level of mesoscutum; metanotum slightly convex, rugose (Fig. 29); propodeum largely punctate-rugose, becoming smooth posteriorly (Fig. 29); posterior propodeal carina distinctly arched, narrow lamelliform, foramen comparatively narrow (Fig. 29) and as high as wide basally.

***Wings.*** Fore wing: vein 1-M 1.1 × as long as vein 1-SR (Fig. 27); second submarginal cell 1.3 × as long as third cell.

***Metasoma.*** First tergite 0.4 × as long as apically wide, smooth and with shallow but wide depression medially (Fig. 31); second tergite largely smooth and shiny medially, moderately punctate laterally; following tergites moderately punctate (Fig. 31); second sternite slightly convex, densely punctate (Fig. 32); third sternite without depression, densely punctate; following sternites densely punctate.

***Colour.*** Black; outer orbita with pale yellow stripes, inner orbita with small patches near malar space (Figs 13, 14); mandibles largely dark brown, with basal patches; pair of elongate patches on middle lobe of mesoscutum anteriorly, pair of patches on antero-lateral margin of scutellum, two pairs of transverse patches on metanotum; palpi, and tegulae dark brown; posterior margin of tergites, 1^st^ and 2^nd^ sternites with ivory stripes (Fig. 33); legs mainly black with tarsi dark brown; pterostigma nearly black; apical half of marginal cell of fore wing largely infuscate as area below it, remainder of wing membrane subhyaline (Fig. 27).

**Male.** Unknown.

#### Biology.

Unknown.

#### Distribution.

China (Tibet). Collected at 2256–2268 m.

#### Etymology.

The specific epithet originates from Greek “eurys”, wide, with reference to the wide terga of metasoma. Treat as a noun in apposition.

### Taeniogonalos taihorina

Taxon classificationAnimaliaHymenopteraTrigonalyidae

(Bischoff, 1914)

FEF16100-3A10-5E81-9BF7-0004267D1EB3

Nanogonalos taihorina Bischoff, 1914: 93; Tsuneki 1991: 58; Weinstein and Austin 1991: 421.
Taeniogonalos taihorina : Carmean and Kimsey 1998: 68; Chen et al. 2014: 171–179 (synonymy, diagnosis, distribution).
Poecilogonalos maga Teranishi, 1929: 148; Marshakov 1981: 106; Tsuneki 1991: 51; Weinstein and Austin 1991: 423; Lelej 1995: 14; Tan et al. 2017: 54–55 (synonymy, distribution)
Taeniogonalos maga : Chen et al. 2014: 146–150 (synonymy, diagnosis, distribution).
Taiwanogonalos claripennis Tsuneki, 1991: 38. Synonymized by Carmean and Kimsey 1998 with T. maga; Tan et al. 2017: 54–55 (synonymy, distribution)

#### Material examined.

1 male, China: Tibet, Motuo, 2954 m, 9.vii.2013, Zhen Liu, 201300003 (ZJUH); 2 males, China: Tibet, Bomi, 3083 m, 12.vii.2013, Zhen Liu, 201300014, 201300015 (ZJUH).

#### Distribution.

China (Fujian, Gansu, Guangxi, Heilongjiang, Ningxia, Shaanxi, Sichuan, Taiwan, Tibet, Yunnan, Zhejiang). Japan (Hokaido, Honshu). Collected at 1200–3083 m.

### Teranishia

Taxon classificationAnimaliaHymenopteraTrigonalyidae

Tsuneki, 1991

13CF592F-2DB5-50EF-A6B1-D24C89C2A360

Teranishia Tsuneki, 1991: 15–18; Lelej 1995: 12, 2003: 3; Carmean and Kimsey 1998: 73; Chen et al. 2014: 193–201 (diagnosis, key to species). Type species (by monotypy): Teranishia nipponica Tsuneki, 1991.

#### Diagnosis.

Antenna black and with 24–27 segments; male antenna without tyoloids; area above supra-antennal elevations flat, more or less punctate, with protuberance between elevations and inner side of supra-antennal elevations flat, smooth and black; occipital carina widened medio-dorsally; apical segment of labial palp widened and obtuse, more or less triangular; vertex normal, at most with slight median depression; mandibles wide in anterior view and sublaterally attached to head; anterior propodeal sulcus distinctly crenulate; metanotum strongly convex and finely sculptured medially; anterior propodeal sulcus crenulate and medially widened; posterior propodeal carina curved and distinctly protruding and more or less separated from foramen medio-dorsally; fore wing with large dark patch below pterostigma; vein 1-SR of fore wing long; hind trochanter black, dark brown or ivory; hind tarsus slightly or not modified; second and third sternites of female flat and moderately sclerotized and no protuberances; body without pale pattern, at most malar space and margins of basal metasomal sternites and tergites narrowly ivory, remainder black (Chen et al. 2014).

#### Biology.

Unknown. Collected in June–September.

#### Distribution.

China, Japan. Chen et al. (2014) reported two species from China. *Teranishia crenulata* Chen, van Achterberg, He & Xu, is newly recorded from Tibet in this study.

### Teranishia crenulata

Taxon classificationAnimaliaHymenopteraTrigonalyidae

Chen, van Achterberg, He & Xu, 2014

5471C2B3-A00F-5145-8CFC-0F1EF7A56C09

Teranishia crenulata Chen et al., 2014: 194–197 (diagnosis, description).

#### Material examined.

3 males, China: Tibet, Motuo, 12.vii.2013, Zhen Liu, 201300011–201300013 (ZJUH).

#### Distribution.

China (Gansu, Ningxia, Sichuan, Tibet). Collected at 1800–2539 m.

## Supplementary Material

XML Treatment for Jezonogonalos

XML Treatment for Jezonogonalos jiangliae

XML Treatment for Jezonogonalos nyingchiensis

XML Treatment for Orthogonalys

XML Treatment for Orthogonalys elongata

XML Treatment for Taeniogonalos

XML Treatment for Taeniogonalos bucarinata

XML Treatment for Taeniogonalos eurysoma

XML Treatment for Taeniogonalos taihorina

XML Treatment for Teranishia

XML Treatment for Teranishia crenulata
